# Prevalence and burden of chronic pain in older adults

**DOI:** 10.1093/geroni/igaf122.2416

**Published:** 2025-12-31

**Authors:** Gillian Fennell, Sarah Tilley, Sayali Dhamne, Emelia Benjamin, Margaret Clancy, Mary Gheller, Robert Edwards, Tuhina Neogi

**Affiliations:** Boston University - Chobanian & Avedisian School of Medicine, Boston, Massachusetts, United States; Boston University - Chobanian & Avedisian School of Medicine, Boston, Massachusetts, United States; Boston University - Chobanian & Avedisian School of Medicine, Boston, Massachusetts, United States; Boston University Chobanian & Avedisian School of Medicine, Boston, Massachusetts, United States; Boston University Chobanian & Avedisian School of Medicine, Boston, Massachusetts, United States; Boston University, Boston, Massachusetts, United States; Brigham and Women’s Hospital, Boston, Massachusetts, United States; Boston University Chobanian & Avedisian School of Medicine, Boston, Massachusetts, United States

## Abstract

Over 100 million Americans experience chronic pain (i.e., pain lasting three or more months), with older adults being disproportionately affected. However, the pattern of pain locations and impact of chronic pain in much older adults have not been well-studied. Further, chronic pain itself has been associated with higher risk of mortality; thus, surviving older adults may report less pain. Greater extent of pain locations, characterized by chronic multisite (≥ 3 pain sites) and chronic widespread pain (WSP, bilateral pain above and below the waist and in the spine), is associated with worse quality of life (QOL) and more severe and activity-interfering pain. Whether these patterns hold true for much older adults is unclear. Using a large, cross-sectional, community-based sample of older adults from the Framingham Heart Study (N = 1,584; mean age, 76.1 ± 7.5; range 52-102 years), we estimated the prevalence and burden of several chronic pain phenotypes by age and sex. Overall, 36.0% had chronic multisite pain, 15.6% chronic WSP, and 10.4% bothersome/high-impact chronic pain (B-HICP) as defined by a combination of pain frequency, severity, and interference. The prevalence of extensive (i.e., multisite or WSP) pain and number of pain sites did not increase with age in men or women. However, pain interference was higher for women and the physical component of HRQOL higher for both men and women with advancing age. In summary, while extensive pain classifications were not more prevalent at older ages, pain burden was positively associated with age.

